# In Search of a Common European Approach to a Healthy Indoor Environment

**DOI:** 10.1289/ehp.8991

**Published:** 2007-01-25

**Authors:** Olaf C.G. Adan, Julie Ng-A-Tham, Wojtek Hanke, Torben Sigsgaard, Peter van den Hazel, Felicia Wu

**Affiliations:** 1 The Netherlands Organisation for Applied Scientific Research TNO, Delft, the Netherlands; 2 Ministry of Housing Spatial Planning and the Environment—DG-Environment, The Hague, the Netherlands; 3 The Nofer Institute of Occupational Medicine, Lodz, Poland; 4 Aarhus University, Department of Environmental & Occupational Medicine, Aarhus, Denmark; 5 Public Health Services Gelderland Midden, Arnhem, the Netherlands; 6 Department of Environmental and Occupational Health, University of Pittsburgh, Pittsburgh, Pennsylvania, USA

**Keywords:** European Union, fragmentation, health, indoor environment, open coordination, policy, subsidiarity

## Abstract

Increasingly, policymakers in Europe and around the world are realizing the importance of healthy indoor environments for public health. Certain member states of the European Union (EU) have already achieved successes in improving indoor environmental quality, such as controlling certain contaminants (e.g., environmental tobacco smoke) or developing nationwide policies that address indoor air generally. However, a common European approach to achieving healthy indoor environments is desirable for several reasons including providing a broader recognition of the problem of unhealthy indoor air, setting a policy example for all 27 EU member states, and achieving greater public health equity across the different European nations. In this article we address the question “Why is it so difficult in the EU to develop a coherent approach on indoor environment?” We identify and describe four main barriers: *a*) the subsidiarity principle in EU policymaking, introducing decentralization of decision making to the member states; *b*) fragmentation of the topic of the indoor environment; *c*) the differences in climate and governance among different member states that make a common policy difficult; and *d*) economic issues. We discuss potential lessons and recommendations from EU and U.S. successes in achieving healthier indoor environments through various policy mechanisms.

## The Link between Indoor Environments and Health Effects

People spend most of their lives indoors, especially at home. On average they spend about 16 hr/day during the week and 17 hr/day during the weekend inside their homes. For children and elderly people, the figures are even higher, ranging from 19 to 20 hr/day and appear to be increasing [World Health Organization (WHO) 2003a].

Early findings indicate a link between housing conditions and human health and well-being. Bonnefoy et al. (2003) have conducted a pan-European survey of several key indoor dwelling characteristics that affect human health: thermal comfort, lighting, moisture, mold, and noise (Table 1). These indoor characteristics can lead to a variety of respiratory diseases, depression and anxiety, and accidental injuries. Excess winter mortality is connected to poor thermal insulation, and to fuel “poverty.” These factors account for an increase in respiratory and cardiovascular ailments. In addition, specific substances found in the housing environment, including asbestos, radon, lead, molds, and volatile organic chemicals (VOCs), have a profound effect on the health of residents (Braubach and Bonnefoy 2001; Fuller-Thomson et al. 2000; Gravesen et al. 1999; Ranson 1991; Reijula 1998; Schwartz 1994). Asthma development and exacerbation have been linked with indoor exposures to animal dander, mold and moisture, cockroach, environmental tobacco smoke (ETS), and house dust mite [Institute of Medicine (IOM) 2000]. These contaminants also may have a role in hypersensitivity pneumonitis disease and chronic obstructive pulmonary disease (COPD; IOM 2004). ETS and radon exposure in indoor environments are both linked with lung cancer (National Research Council 1999).

Although the indoor environment has attracted the interest of scientists in the public health sector since ancient times (Foster 1992; Ineichen 1993; Krieger and Higgins 2002), more work is needed on the policies concerning this issue in order to ensure healthy indoor environments throughout Europe. The problem of housing has become even more important since World War II. Social housing (i.e., subsidized housing for low-income people) across the 27 member states of the European Union (EU) includes more than 55 million dwellings. Much of this housing is of poor quality, creating health problems and insecurity for the occupants and ongoing maintenance problems for the owners. At present, more than 170 million people live in mass housing constructed in the postwar period.

Many countries in Western Europe are undergoing rapid decentralization, and as a result, local authorities have been given increased responsibility for housing. Further, the political and economic situations that have emerged in Central and Eastern Europe since the fall of the Berlin Wall have created dramatically new housing situations. In some countries the percentage of homeowners has increased to more than 95% (Haffner and Doll 2000). Because state organizations are not maintaining private property, an accelerated deterioration of the housing stock is taking place, with an increasing number of poor indoor environments.

The age of the population is increasing. In Europe the percentage of adults older than 65 years will increase from 15.5% in 2000 to 19.6% in 2020 (Doll and Haffner 2001). Care of the elderly and social inclusion are increasingly important problems from a housing standpoint. Life expectancy, on average, has risen in Europe from 50 to 80 years in less than a century, while during the same period the housing stock has been renewed by only 60%. Aware of these trends, the WHO Regional Office for Europe embarked on a study to review and enlarge the body of evidence regarding the relationship between housing conditions and health. This pan-European study took place in seven cities across Europe (Bonnefoy et al. 2003; WHO 2003a). The goal of the survey was to provide a basis for a common set of priorities to achieve the greatest health gain in housing rehabilitation programs. The aim continues to be to guide policymaking at both the local and community levels to this end, with final results expected in 2007.

Although the concentrations of major indoor air pollutants may be different in the Nordic countries compared with the southern area of Europe, the main indoor environmental issues affect most European countries and therefore should be prominent on the EU agenda. However, until recently little interest was expressed in this topic and in developing a common European policy. In this article we address the question “Why is it so difficult in the European Union to develop a coherent approach on indoor environmental quality?” We begin by describing why a common EU approach would be beneficial and describe EU-wide initiatives that are now taking place to improve indoor environments. We then discuss potential barriers to EU-wide indoor air policy development. Finally, we give recommendations for EU-wide policy development, in part based on policy successes elsewhere.

## The Need for a Common European Approach to Healthier Indoor Environments

There are several reasons that a common EU approach to achieve healthier indoor environments is desirable. The first is to provide greater recognition of the indoor environmental quality (IEQ) problem throughout Europe. Although several nations already recognize the importance between indoor environments and public health, as indicated in the policies they have developed, others have no such policies, and IEQ may not be high on their agendas despite the crucial role it plays in the health of their citizens. A common EU approach would highlight the importance of IEQ and bring it to the attention of national and local policymakers throughout Europe.

Second, a common EU policy approach would provide an example for individual member states and local governances to establish additional and more coherent national policies to improve IEQ. At present, such coherence is lacking.

Third, linked with these two issues is the importance of achieving greater health equity across the 25 EU member states through a common EU IEQ policy. As the EU continues to expand, one of the most pressing issues is how to achieve a more uniform, higher quality of living for all EU citizens. Tackling the IEQ problem from a common perspective may be one of the most efficient ways to ensure greater equity in terms of health outcomes such as respiratory disease and well-being related to housing, workplaces, and public buildings.

## Current EU Initiatives toward Healthier Indoor Environments

The need to address the indoor environment at the EU level is recognized by the EU (Commission of the European Communities 2004) and WHO (2004). For EU-wide indoor environments, both specific and general expertise is needed in multiple areas. This IEQ problem can provide the onset of policy, using the “open method of coordination.” The method involves transparency and sharing knowledge and experience, which can lead to better consistency in national policies and regulation (Borrás and Jocobsson 2004; Radaelli 2003; Treib et al. 2005) This “bottom-up” approach can be used when there is no clear mandate for a legally based policy. In the EU it is an alternative or an addition to the top-down “community method” used by the European Council and the European Parliament to pass regulations proposed by the European Commission (Radaelli 2003). Member states now feel the urgency to share knowledge and experience on the indoor environment. WHO and the European Commission and member states all use these mechanisms.

Several initiatives are currently under way within the EU to achieve healthier indoor environments. Below, we present these initiatives together with the main challenges for their success.

### Efforts toward controlling environmental tobacco smoke in Europe

The WHO European Strategy for Tobacco Control (ESTC; WHO 2002) was first implemented in late 2002. The WHO Framework Convention on Tobacco Control (FCTC; WHO 2003b) has reinforced the fight against the tobacco epidemic, opening new opportunities and challenges in this field. Many EU member states have strengthened their tobacco control policies and legislation in line with the ESTC and FCTC in recent years. Challenges, however, remain in other countries and areas of concern, and much can be achieved by the review of recent developments, practices, and lessons learned.

At the initiative of the EU, member states have agreed to take individual measures to control ETS (EU 2005). By April 2006, six countries—Ireland, Norway, Italy, Malta, Scotland, and Sweden—had introduced smoke-free bars and restaurants, and England will follow suit shortly. A complete ban on smoking in the workplace, excluding bars and restaurants, and with the possibility of designated and ventilated smoking rooms has been implemented in Finland, Iceland, the Netherlands, and Belgium. In addition, many countries in Europe have legislations that provide smoking zones and areas either in the workplace or in bars and restaurants (European Respiratory Society 2006).

### Joining forces in the European Construction Technology Platform (ECTP): stakeholders’ collaboration toward a research agenda

In 2004 the European Commission and industry proposed the launch of European technology platforms. These platforms were initiated to bring together companies, research institutions, the financial world, and regulatory authorities in Europe to define a common research agenda. Such joining of forces would mobilize a critical mass of national and European public and private resources. Specifically, the European public relies on the construction sector to obtain better living and working conditions for its built environment. The formation of the ECTP is one of the initiatives responding to the Commission’s proposal. It brings together more than 300 committed stakeholders at all levels of the supply chain, including client organizations and public bodies.

On the basis of their vision for 2030, the members of the ECTP drafted the first strategic research agenda (ECTP 2005), paving the way for future research by setting out the likely directions and priorities. The ECTP believes construction is increasingly client driven and proposes two interlinked key goals: first, becoming sustainable as the driver for development, and second, meeting client requirements. Health, comfort, and safety in the built environment are considered the key client requirements for success. The research goals and ambitions typically address development of new (design and production) concepts and new materials that create:

- an accessible and safe indoor environment for all individuals (including semienclosed spaces such as open stations and the immediate outside area of buildings);
- a 50% reduction in the number of accidents that occur home;
- a 20% reduction of sick building syndrome; and
- a reduction of 20% of people who suffer from asthma, allergies, and other respiratory diseases.

Importantly, in ECTP’s priorities, the end user (the citizen or the client) has a pivotal position. The examples are self-evident. The early and active involvement of the European Disability Forum, which is an umbrella organization representing more than 50 million disabled people in Europe, resulted in a clear set of common objectives ensuring disabled citizens full access in the built environment. The direct and indirect interactions with public organizations such as WHO and those involved in health care led to clear objectives in terms of end-user requirements, for example, the 2030 target date to reduce household accidents. This should be considered a major change in a sector that is not only highly fragmented but that also traditionally has a technology-driven orientation toward construction of products.

The impact of the construction sector on society gives the ECTP an important European dimension. The continuous stakeholder interaction in the triangle comprising industry, research, and the public has introduced a mutual understanding of interests and priorities, including the issues of indoor environment and public health and safety. This bottom-up approach seems to tackle simultaneously both market and knowledge fragmentation and encourages the formation of new public–private partnerships and alliances.

Industry’s involvement of the consumer in the definition of the strategic research agenda of the construction area appears to be a major step forward, making the indoor environment a priority of the sector. The strong commitment from industry is likely to guarantee that innovation, including health-related innovations, will find its way to practical implementation. Still, real progress toward identifying long-term priorities and needs is not an easy task, as market forces rather than the vision of tomorrow usually drives research and development in construction. The challenge is to turn the cautious change sketched above into a lasting one, even without the strong financial incentive of EC funding.

## EU Networks: Assembling Knowledge from Research and Delivering to Policymakers

The EU-initiated thematic networks may be an effective instrument for development of European research that produces and delivers findings to policymakers. At present, the Policy Interpretation Network on Children’s Health and Environment (PINCHE) is a running thematic network funded by the Directorate General Research, in the Quality of Life and Management of Living Resources (LIFE) Programme of the Fifth Framework Programme (Bolte and Kohlhuber 2005; Van den Hazel and Zuurbier 2005; Van den Hazel et al. 2005a, 2005b, 2005c; Zuurbier and Van den Hazel 2005a, 2005b). This network unites researchers, representatives from industry, consumer and patient organizations, and nongovernmental bodies.

The main objective of PINCHE is to provide the EU with policy recommendations for protecting the health and environment of children based on results of scientific studies, both EU-funded and other national studies on environment and health. PINCHE focuses on children, a vulnerable population largely ignored by present day legislation that is concerned primarily with the adult population (Tamburlini et al. 2002). This vulnerability is caused both by children’s greater susceptibility resulting from both their immature organ systems and differences in their exposures (e.g., foods consumption) and exposure time (Charnley 2001; Dalston et al. 2004; Gee 1999; National Research Council 1993; Tamburlini G, unpublished data; Tamburlini et al. 2002; U.S. Environmental Protection Agency 2003).

Regarding indoor air exposures, the network brings together knowledge on a wide range of pollutant sources including ETS, allergens, molds, organic dust (endotoxins), formaldehyde and other volatile organic compounds, chlorination by-products and cleaning products, polycyclic aromatic hydrocarbons (PAHs), bromated flame retardants, pesticides, radon daughters, and finally, asbestos. In addition, all stages of life are considered, including prenatal exposure and preconception exposure for compounds that accumulate in the mother’s body and compounds that influence the semen. PINCHE focuses on the “EU-27 region” (all 27 members of the EU) but intends to widen the scope of its policy implications to associated countries and potential candidate countries.

Results of PINCHE indicate bottlenecks in the thematic network approach. Three main challenges for success have been identified. First, is data comparability (European Commission 2004). PINCHE identified the need for standardization of *a*) environmental assessments, including estimates of ETS exposure, of indoor and outdoor air quality (Kollar and Mücke 2000), and of dietary (including breast feeding) and exercise habits and practices; *b*) classification of childhood respiratory diseases and symptoms; and *c*) a format for defining diagnostic groups and presentation of data. Furthermore, the importance of morphologic data on cancers in children and young people was recognized.

Second, data accessibility must be addressed (European Commission 2004). Although the internet is a powerful tool for rapid access of information and data, publication of scientific data usually lags 1–2 years behind completion of a study. The subsequent step, accessibility of the scientific data to the general public including health professionals and policymakers, is even more crucial and requires a translation that often is lacking.

Finally, there is a requirement to standardize definitions and methods to ensure that scientists and authorities speak the same language (European Commission 2004). This is crucial to a viable science–policy interface. As with all networks, there is the inherent threat of becoming “self-protective.” A closed network becomes self-persistent, thereby remaining too small and having limited research translated into policy recommendations. Networks should remain open, but integration should not expand too widely, as that may also lead to a weakened identity and reduced impact. The challenge is to find the balance.

## Barriers to Developing EU-Wide Policies on Healthy Indoor Environments

A variety of barriers exists, however, to the development of EU-wide policies to achieve indoor environmental quality. Here we discuss four categories of barriers: the subsidiarity principle, fragmentation of expertise and purpose at various levels, the important climatic and governance differences among the EU member states, and economic issues.

Among the barriers that prevent indoor environmental quality from reaching the EU policy agenda is the subsidiarity principle. This principle, common throughout EU regulatory decision making, states that policy action will be taken at the EU level only when it would be more effective than action taken at a national, regional, or local level. This principle is applied when both the EU and its member states have competence in a particular policy area, as is the case with the indoor environment. In such cases, the subsidiarity principle ensures decentralization, allowing member states to have their own policies. While this may allow for individual member states, or even individual regions within a member state, to develop IEQ policies that are well suited to their unique situation, the subsidiarity principle also makes it more difficult for the EU to provide guidance and expertise where national or local IEQ policies have not been developed. The risks of overregulation make it difficult to challenge the subsidiarity principle.

A second barrier to developing an EU policy is fragmentation. This may occur in the areas of disciplines and expertise, interests, and responsibilities. At the discipline level, there is fragmentation of expertise in science, engineering, and health. The indoor environment encompasses such disparate topics as temperature, air quality, radiation, noise, and health effects, each with its own experts. For example, constituents of indoor air range from volatile to persistent organic chemicals, to biologics such as molds, and to particulates. As such, the expertise needed to address indoor environmental quality is scattered among diverse research fields and disciplines. This fragmentation of knowledge, inherently linked to the multidisciplinary nature of health and the indoor environment, makes it difficult to create an integrated comprehensive knowledge base that is needed to set an agreed-upon agenda for policymaking.

At the stakeholder level there is a fragmentation of interests. The stakeholders in the indoor environment vary from builders, architects, raw material suppliers, producers of building materials and interior decorating materials, to owners and occupants. Often these stakeholders do not live with the consequences of their decisions, so there is little interest in ensuring a healthy indoor environment (Wu et al. 2006). Moreover, different stakeholders have specific (national or local) interests, making a European approach difficult. Health interests are often neglected in a market traditionally pushed by technology. However, recognition of the need for a more unified approach is growing.

Also, there is fragmentation of responsibilities at the level of regulatory authorities and policymakers. This makes it difficult to find a protagonist who will push indoor environmental quality up the political agenda. At both the EU and the member state level, typically there is more than one department responsible for the area of IEQ, for example, the departments of housing, public health, environment, and trade. Therefore, many responsible parties need time to decide to pursue the subject and coordinate the various views and responsibilities. Because fragmentation is faced at all policy levels (local, national, and EU levels), much deliberation and coordination is needed to reach the EU policymaking agenda.

A third barrier is the difficulty of uniting such disparate climates, social factors, and governances under a common EU policy regarding indoor environments. The 25 member states of the EU span a variety of climates, from polar to almost subtropical and from very dry to very wet. This variety can have an important effect on the types of indoor environmental contaminants that are relevant to different member states. For example, those living in more humid regions in the EU are more susceptible to the adverse health effects caused by mold and moisture in indoor environments, whereas those living in dryer regions may suffer little or not at all from these indoor problems. To develop EU-wide policies on this one IEQ problem alone, in addition to a multitude of potential IEQ problems, would therefore be very difficult.

Moreover, among the factors that affect indoor environmental quality are human behavior, household products, and climate control systems. Certain crucial factors affecting IEQ, such as ventilation (referring to the ventilation system, as well as to use and quality control), depend on regional and social differences such as the warmth and humidity of the region, the quality of the housing, and the reliability of maintenance systems in that region. Differences in governance in different member states and regions would affect whether any EU policy on IEQ would be sufficiently enforced to ensure adequately healthy indoor environments. Some member states already have IEQ policies in place, whereas in other member states, indoor air issues do not receive much attention and their policies are not well developed.

A final barrier to a common EU approach to indoor environments is economics. A common EU approach may invite overregulation on an issue for which economic incentives of individuals would not align with the goal of an “ideal” indoor environment. As an example, individuals furnish and maintain their indoor environments often based on cost-based rather than health-based decisions. Ventilation rates in private European houses are commonly determined by high energy costs rather than EU-wide regulations; thus, the value of its regulation is debatable. To a certain extent, even in the context of health effects resulting from IEQ, individuals should be allowed to choose for themselves what is more important to them—cost savings or a highly healthy indoor environment—rather than for a common EU approach to impose regulations on such choices.

## Policy Recommendations

These issues and barriers described above are not relevant only to the EU but also to other parts of the world. In the United States, for example, there are similar barriers to erecting U.S.-wide policies to improve indoor environments. Wu et al. (2007) describe these barriers and also address socioeconomic issues and differences in multiple stakeholders’ interests. The authors provide recommendations for realigning economic incentives and disseminating information, which could affect some of the barriers discussed above.

For example, fragmentation of interests and responsibilities can be addressed by providing certain stakeholder groups—architects, building designers and construction staff, and landlords—with awards or special labels to encourage them to design buildings that meet a specified level of indoor environmental quality. Benefit–cost analyses of the costs associated with poor indoor environments, and the benefits of healthier ones, will provide incentives for policymakers to make IEQ issues a priority, especially if these values can be expressed in economic terms.

Jacobs et al. (2006) describe U.S. successes in regulation of lead and radon in indoor environments, which can provide further lessons for the EU in developing a common IEQ policy. For example, although the term “subsidiarity” is not typically used in the United States to describe division of responsibility among the federal, state, and local governments, many of the concepts continue to apply for indoor air. Lead, in part because of its former ubiquity in homes (primarily through paint) and fuel throughout the United States as well as its well-defined health effects, is regulated at the federal level through a variety of laws limiting human exposure. Radon, on the other hand, is regulated at state and local levels in part because radon levels in the ground, and hence in buildings, differ substantially throughout the United States.

The EU might learn from these U.S. policies in order to divide governance of prominent indoor environmental hazards among the proper regulatory bodies—EU, member state, or local—depending on differences in exposure to the hazard. This would solve some of the problems associated with both subsidiarity and disparities in exposure. For example, an indoor air problem that is ubiquitous across EU member states could be regulated at the EU level (e.g., ETS), whereas indoor air problems (possibly mold) that are more prominent in certain areas of Europe than in others could be regulated at state and local levels.

As for how disparities among different governing bodies can be united to the common cause of improving IEQ, we present one example of IEQ policies in one EU member nation, Denmark, where four relevant ministries divide or share responsibilities related to indoor air.

### Indoor environmental quality policies in Denmark

The policy of the Danish government is that good quality indoor air should be maintained, and health risks related to indoor air should be reduced to the lowest possible level. In this context, dissemination of knowledge and best practices to the general public and stakeholders in particular is crucial.

Four ministries in concert address regulation of indoor air quality (IAQ): the Ministry of Economic and Business Affairs, the Ministry of Environment, the Ministry of Employment, and the Ministry of Interior and Health. Each has a specific responsibility for defined indoor application areas, ranging from private housing to public buildings such as schools and hospitals. The ministries are involved simultaneously and share responsibilities from different viewpoints. For example, for schools, the Ministry of Employment is responsible for school employees, and the Ministry of Economic and Business Affairs is responsible for the pupils.

Ministry policies are based primarily on research results produced by their own agencies. The Danish Building Research Institute is the agency for The Ministry of Economic and Business Affairs; the Working Environment Authority is run by the Ministry of Employment; the National Board of Health is the agency for the Ministry of Interior and Health; and the Danish Environmental Protection Agency (DEPA) is the agency for the Ministry of Environment. In addition to supporting policymaking, each agency has a specific area for dissemination of knowledge.

The Danish Building Research Institute (SBi 2006) provides guidance to the construction industry on an ad hoc basis. It regularly produces practical guidelines on indoor climate control, of which “The Indoor Climate Guide” (Valbjørn et al. 1995) is considered the most important. This guide provides an overview of guidelines for architects, engineers, building contractors and building owners in planning nonindustrial buildings such as kindergartens. The SBi also developed guidelines for homeowners and tenants, explaining how to detect and avoid dampness problems in their homes.

The National Board of Health (2006) is responsible for public health and focuses on emissions from mineral wool, asbestos, carpets, chemical pollutions, molds, and radon in the context of indoor air quality. The board addresses particularly schools, kindergartens, and other public buildings.

The Working Environment Authority (WEA 2006) focuses on indoor air quality of workplaces. The WEA has created a home page where companies can download an IAQ questionnaire to assess complaints in offices, schools, and kindergartens, among other workplaces.

## Summary

The indoor environment is crucial to our health and well-being, as we in the western world spend the vast majority of our time indoors. Indoor environmental contaminants are associated with a variety of respiratory and other diseases and symptoms that range from asthma to depression to lung cancer. Throughout Europe, particularly in the years following World War II, housing quality has become an increasingly important issue. There are a number of reasons why a common EU approach to addressing healthy indoor environments would be desirable, among them, to increase the recognition of the importance of this health problem and to provide a policy framework for individual member states.

Considering the importance of indoor environments, it would be expected to figure prominently in the European policy agenda. However, this is not the case. There are several examples of IEQ successes achieved in individual member states, such as the case of ETS in public spaces in six EU nations. However, at the EU-wide level, a more integrated policy approach is needed. Only recently has indoor air, one aspect of the indoor environment, made its cautious appearance in the WHO–Euro ministerial declaration (WHO 2004) and the EU Environment and Health Action Plan (European Commission 2004). Although this EU starting point was welcomed and encouraged by the member states (Anonymous 2004) and the European Parliament (Committee on the Environment Public Health and Food Safety 2005), the initiated process is moving slowly and remains quite invisible.

Several initiatives have begun to pave the way toward a common EU approach to IEQ. The EU and member states are using aspects of the open method of communication with varying degrees of success to counter these barriers. The ECTP approach uses the combination of the bottom-up approach with financial incentive, which seems to tackle simultaneously both market and knowledge fragmentation. Presently, both the strong commitment and firm involvement of the consumer in the ECTP appears to be a big step forward, making the indoor environment a priority of the sector. The challenge now is to surpass this initial stage of “good intentions” induced by external pressure and stimuli and to begin to act on the intentions. External feedback mechanisms and critical external auditing, coupled with the financial incentive, remain necessary to stimulate action. Cooperation must also be stimulated. To ensure this interaction, attention should focus on feedback and reward mechanisms. Because a large body of knowledge exists on issues of motivation and pay and performance management, we point to the possible usefulness of this knowledge in the design and underpinning of feedback and reward mechanisms.

PINCHE is a valuable step toward the creation of a widely supported solid scientific basis for public health policymaking to protect the health and environment of children. The network has succeeded in bringing together scientists from a wide variety of disciplines. The next steps of data comparability and data accessibility are critically important. The science–policy interface that the network is committed to developing depends on successfully summarizing and updating the available knowledge for policymaking and in so doing, acquiring an undisputed scientific reputation. During the limited time that the network has existed, it is still working to establish this interface. This will remain the challenge. Obtaining funding to continue the work will be important. Although stimulation of networks is clearly a step in overcoming the barrier of fragmentation, much remains to be done to overcome the fragmentation.

The topic of cooperation has reached the EU agenda. Although the EU member states, WHO, and the European Commission have each initiated activities to foster cooperation, they have not succeeded in finding the most fruitful way to incorporate the various activities and roles concerning the indoor environment. As the players are still scattered in their responsibilities concerning indoor environments, it will be a challenge to coordinate and better utilize the ongoing activities of each of the parties. A dialogue on an overview of the ongoing activities, both at the EU-wide and member-state levels, could be a useful first step.

Despite the progress already made, developing a coherent approach on the indoor environment is still difficult in the EU. We have identified and described four main barriers: *a*) the subsidiarity principle in EU policy-making, which introduces decentralization of decision making to the member states; *b*) fragmentation of the topic of the indoor environment; *c*) the differences in climate and governance among different member states that make a common policy difficult; and *d*) economic issues. Lessons from successes in the EU and United States in achieving healthier indoor environments through various policy mechanisms may provide useful insights into achieving EU-wide policy successes in indoor environmental quality.

## Figures and Tables

**Table 1:** Preliminary trends in the relationship between housing and health, in the WHO pan-European survey.^a^

Physical parameter	Dissatisfaction	Human health: determined links
Temperature comfort	50% (highly) dissatisfied with thermal multiple allergies	Respiratory diseases, cold and throat illness
Light	25% people (highly) dissatisfied (daylight)	(Trends of) depression, chronic anxiety, household accidents
Noise	25% people annoyed (e.g., traffic, neighbors)^b^	Hypertension, (trends of) depression, fatigue, accidents
Moisture and mold	25% of dwellings: mold growth in > 1 room 8% of dwellings: smells, dampness	Respiratory diseases, asthma, allergies^c^
Indoor air quality (general)	10% dissatisfied	Fatigue, (trends of) depression, anxiety, respiratory diseases

a Data from Bonnefoy et al. (2003);

b Sixth Environment and Health Action Plan (Commission of the European Communities 2004); and

c Samson et al. (1994)
